# Updated Role of Neuropeptide Y in Nicotine-Induced Endothelial Dysfunction and Atherosclerosis

**DOI:** 10.3389/fcvm.2021.630968

**Published:** 2021-02-23

**Authors:** Yan-li Zheng, Wan-da Wang, Mei-mei Li, Shu Lin, Hui-li Lin

**Affiliations:** ^1^Department of Cardiology, The Second Affiliated Hospital of Fujian Medical University, Quanzhou, China; ^2^Centre of Neurological and Metabolic Research, The Second Affiliated Hospital of Fujian Medical University, Quanzhou, China; ^3^Diabetes and Metabolism Division, Garvan Institute of Medical Research, Sydney, NSW, Australia

**Keywords:** neuropeptide Y, nicotine, endothelial dysfunction, atherosclerosis, cardiovascular disease

## Abstract

Cardiovascular disease is the leading cause of death worldwide. Endothelial dysfunction of the arterial vasculature plays a pivotal role in cardiovascular pathogenesis. Nicotine-induced endothelial dysfunction substantially contributes to the development of arteriosclerotic cardiovascular disease. Nicotine promotes oxidative inflammation, thrombosis, pathological angiogenesis, and vasoconstriction, and induces insulin resistance. However, the exact mechanism through which nicotine induces endothelial dysfunction remains unclear. Neuropeptide Y (NPY) is widely distributed in the central nervous system and peripheral tissues, and it participates in the pathogenesis of atherosclerosis by regulating vasoconstriction, energy metabolism, local plaque inflammatory response, activation and aggregation of platelets, and stress and anxiety-related emotion. Nicotine can increase the expression of NPY, suggesting that NPY is involved in nicotine-induced endothelial dysfunction. Herein, we present an updated review of the possible mechanisms of nicotine-induced atherosclerosis, with a focus on endothelial cell dysfunction associated with nicotine and NPY.

## Introduction

Smoking is a serious global public health problem and an independent risk factor for cardiovascular disease. Nicotine is the main toxic substance in tobacco. Nicotine can induce endothelial dysfunction, which may lead to pathophysiological states contributing to the development of vascular disorders resulting from atherosclerosis (AS). Although nicotine-induced vascular endothelial dysfunction has been characterized, the mechanism has not been fully elucidated (1). Accumulated evidence has found that after nicotine exposure, the expression level of central and peripheral neuropeptide Y (NPY) changes. For example, *NPY* mRNA expression increased substantially in the hypothalamus of rodents administered the same dose of nicotine as that consumed by ordinary smokers (2, 3). Nicotine-induced noradrenaline (NA) release in perfused guinea pig hearts is accompanied by NPY overflow in the coronary venous system (4). The NPY system is strongly associated with arteriosclerotic cardiovascular disease. The binding of NPY to the Y1 receptor may be involved in the pathogenesis of chronic methamphetamine-induced AS (5). Therefore, NPY regulation plays a decisive role in the development of cardiovascular disease. There is increasing evidence that nicotine can cause disordered blood flow, which can induce endothelial dysfunction. Moreover, NPY can induce blood flow disorders through a variety of pathophysiological changes. NPY and nicotine may play a combined role in promoting endothelial

dysfunction. The correlation between NPY and nicotine exposure-associated endothelial dysfunction and the underlying mechanisms are unknown. This review examines the role of NPY in nicotine-induced endothelial dysfunction, with a focus on the relationship between the nicotine/NPY system and the occurrence and development of arteriosclerotic cardiovascular disease.

## Vascular Endothelial Function

Endothelial cells (ECs) in the heart and vascular system, serve as important barriers between the blood and vascular walls and are innervated by sympathetic and parasympathetic nerves. In addition to playing a vital role in normal angiogenesis, dynamic balance, and vascular tone regulation, the endothelium is also an important metabolic and secretory organ. Endothelial products, including nitric oxide synthases (NOS), hydrogen sulfide, prostacyclin, endothelins, and thromboxane A2 (TXA2), affect the contraction and dilation of human blood vessels (6). NOS, comprising endothelial NOS (eNOS), neuronal NOS (nNOS), and inducible NOS (iNOS) are critical enzymes in nitric oxide (NO) production (7). ECs prevent arteriosclerotic cardiovascular disease by maintaining the delicate balance between hemorrhage and thrombosis by inducing the expression of coagulation factors and anticoagulants such as tissue factor (TF), von Willebrand factor, and fibrinolytic components; enhancing endogenous antioxidant capacity, especially the secretion of eNOS; promoting angiogenesis by secreting angiogenic growth factors, such as vascular endothelial growth factor (VEGF) and fibroblast growth factor; organizing immune cell recruitment by secreting chemokines and adhesion molecules; and transporting nutrients and signals. The physiological function of the circulatory system thus depends on the structural integrity of the endothelium.

## Nicotine and Endothelial Function

Nicotine can increase the release of neurotransmitters, particularly aminergic substances such as NA by stimulating nicotinic acetylcholinergic receptors (nAChR) that mainly act on chromaffin and nerve cells. The physiological form of nicotine not only induces angiogenesis, mediated by nAChR effects on ECs, but also promotes EC mitosis by inducing the secretion of angiogenic factors (8, 9). Nicotine stimulates the production of reactive oxygen species (ROS) that activate scavenger receptors, and ultimately lead to leukocyte adhesion and increased cell permeability. Nicotine does not merely reduce the secretion and bioavailability of NO by promoting eNOS uncoupling and changing the mitochondrial electron transport chain (10), it also affects the secretion of insulin and glucagon, which together lead to EC energy metabolism disruption. Besides increasing vascular tension to change the inner radius of the vessel, nicotine increases blood viscosity by increasing the quantity of plasma components such as inflammatory factors, leukocyte, and coagulation factor. Both the viscosity of blood and the inner radius of vessel can change the magnitude of shear stress, resulting in disturbed flow that induces endothelial dysfunction (11, 12). In essence, nicotine is detrimental to overall endothelial function.

## NPY and Receptors

The 36-amino-acid polypeptide NPY, belongs to the same neuroendocrine peptide NPY family as the pancreatic polypeptide and peptide YY. NPY plays an important role in appetite, anxiety state, angiogenesis, and vasoconstriction, and is widely distributed in the central and peripheral nervous systems, especially in the hypothalamus (13). The NPY-Y receptor system belongs to the G-protein-coupled receptor superfamily; there are at least four receptors in most mammals, namely, Y1, Y2, Y4, and Y5 receptors, which have different affinity and selectivity (14, 15). Although NPY is mainly secreted by sympathetic nerve cells and pheochromaffin cells, it is also present in peripheral nerve terminals, peripheral fat cells, platelets, liver, and ECs (16). Central NPY can be jointly released into the peripheral circulation (17), and is associated with food intake (18, 19) and mood regulation (20). For example, NPY induces an anxiety state through Y2R but alleviates anxiety by binding to Y1R (21, 22). The central NPY system is also closely associated with cardiovascular regulation. NPY has notably emerged as an important transmitter that can bind to different receptors, promote thrombosis, constrict blood vessels, and regulate insulin secretion (23, 24). The characteristics of NPY receptors are summarized in Table 1.

**Table 1:** Characteristics of NPY receptors.

**Species**	**Location**	**NPY receptor**	**Functional effect**	**References**
Animal	Aortic endothelial cell (rat)	Y1R, Y2R, Y5R	Stimulates migration, proliferation, and tube formation	(25)
	Hypothalamus (rat)	Y5R	Induces adipocyte insulin resistance	(26)
	Coronary microvessels (canine)	Unknown	Stimulates vasoconstriction	(27)
	Ischemic tissue, carotid artery, platelet (mice)	Y1R, Y2R, Y5R	Induces ischemia, angiogenesis, neointima, vascular obstruction, atherosclerotic lesion burden, and structural vulnerability	(28–30)
	Hypothalamus (rat)	Y2R	Induces anxiety state	(31, 32)
	Coronary artery (porcine)	Y1R	Stimulates release of TXA2	(33)
	Ventricle, Islet (mice)	Y1R	Inhibits insulin secretion	(34)
	Central nervous system (rat)	Y1R	Stimulates appetite	(35, 36)
	Brain (rat)	Y1R, Y5R	Modulates triglyceride secretion	(37)
	Brain (rat)	Y1R	Attenuates somatic nicotine withdrawal signs	(38)
Human	Peripheral vessels	Y1R	Stimulates vasoconstriction	(39, 40)
	Sympathetic nerve cells, saphenous vein	Unknown	Activates platelets and promotes coagulation	(41, 42)
	Blood vessel	Y1R, Y2R (mainly)	Stimulates sprouting and adhesion, migration, proliferation, angiogenesis	(43)
	Allelotype	Y2R	Reinforces nicotine dependence	(44)

## NPY and Endothelial Function

**Figure 1:**
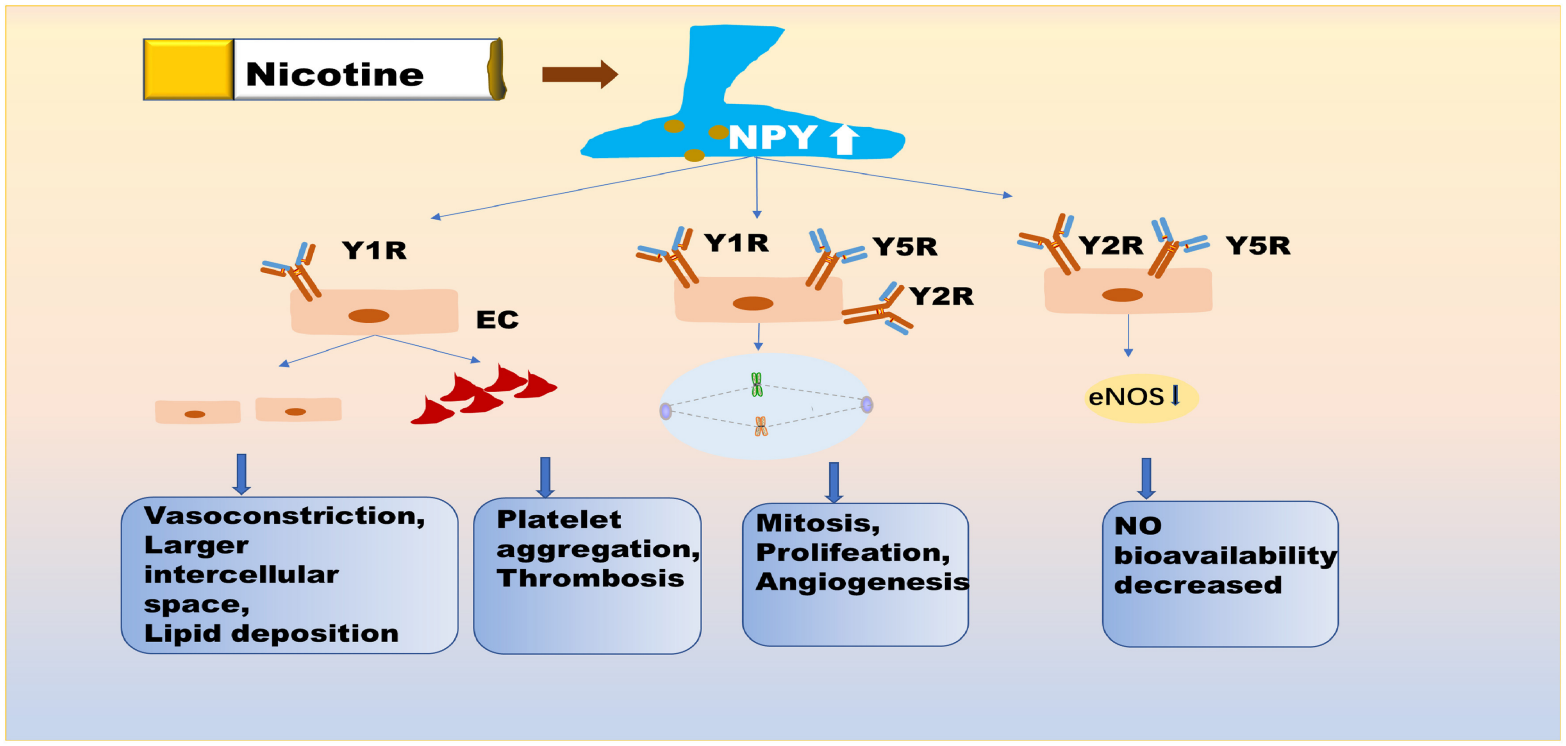
NPY is directly involved in nicotine-induced endothelial dysfunction. Nicotine exposure promotes the expression of NPY in central and peripheral neuronal pathways. NPY not only promotes vasoconstriction and increases intercellular space, resulting in the deposition of lipid in the intima, but it also promotes platelet aggregation and thrombosis via Y1R on EC. NPY further affects the mitotic process, endothelial cell proliferation, and angiogenesis, primarily by binding to Y1R, Y2R, and Y5R. NPY also reduces the secretion of eNOS from endothelial cells, by binding to Y2R and Y5R. Y5R plays the role of enhancer for Y1R and Y2R. These pathological processes affect the normal function of endothelial cells. NPY, neuropeptide Y; Y1R/Y2R/Y5R, Y1/Y2/Y5 receptor; eNOS, endothelial nitric oxide synthase; EC, endothelial cell.

At least three receptors have been identified on ECs, namely, Y1R, Y2R, and Y5R (Figure 1). NPY at the physiological concentrations of eNOS and VEGF, stimulated endothelial cell proliferation, germination, migration, and adhesion, and induced ischemic angiogenesis and intimal thickening (43, 45), by binding to Y1R, Y2R, and Y5R (25, 46, 47). In AS, abnormal neovascularization in plaques not only accelerates plaque progression but also increases the risk of plaque rupture and hemorrhage (48). Vasoconstriction and the discontinuity of ECs might be caused by Y1R activation within the cardiovascular system (49). Comparably, Y1R on macrophages is involved in the inflammatory response, which may contribute to endothelial dysfunction (50). Additionally, Y1R on ECs has a role in the induction of thrombosis. Sympathetic excitement may be associated with the platelet activation state. Endothelial damage stimulates NPY secretion, which induces TXA2 release, and NPY binds to Y1R on ECs, thereby promoting platelet aggregation (41, 51). NPY is a powerful orexin that plays a crucial role in fat storage (33) and is the main source of blood lipids. NPY not only modulates insulin secretion (35, 52), but substantially induces insulin resistance in hepatocytes and adipocytes through Y1R and Y5R (26, 53, 54). Endothelial dysfunction induced by NPY may be reduced NO bioavailability, partly due to Y2R-induced anxiety (55, 56). The Y2R also calibrates peripheral NA secretion (57). Compared to other NPY receptors, the role of Y5R is unclear. A recent study reports that Y5R activity potentiates the function of Y1R and Y2R to promote endothelial cell proliferation (58).

## NPY and Nicotine-Induced Endothelial Dysfunction

**Figure 2:**
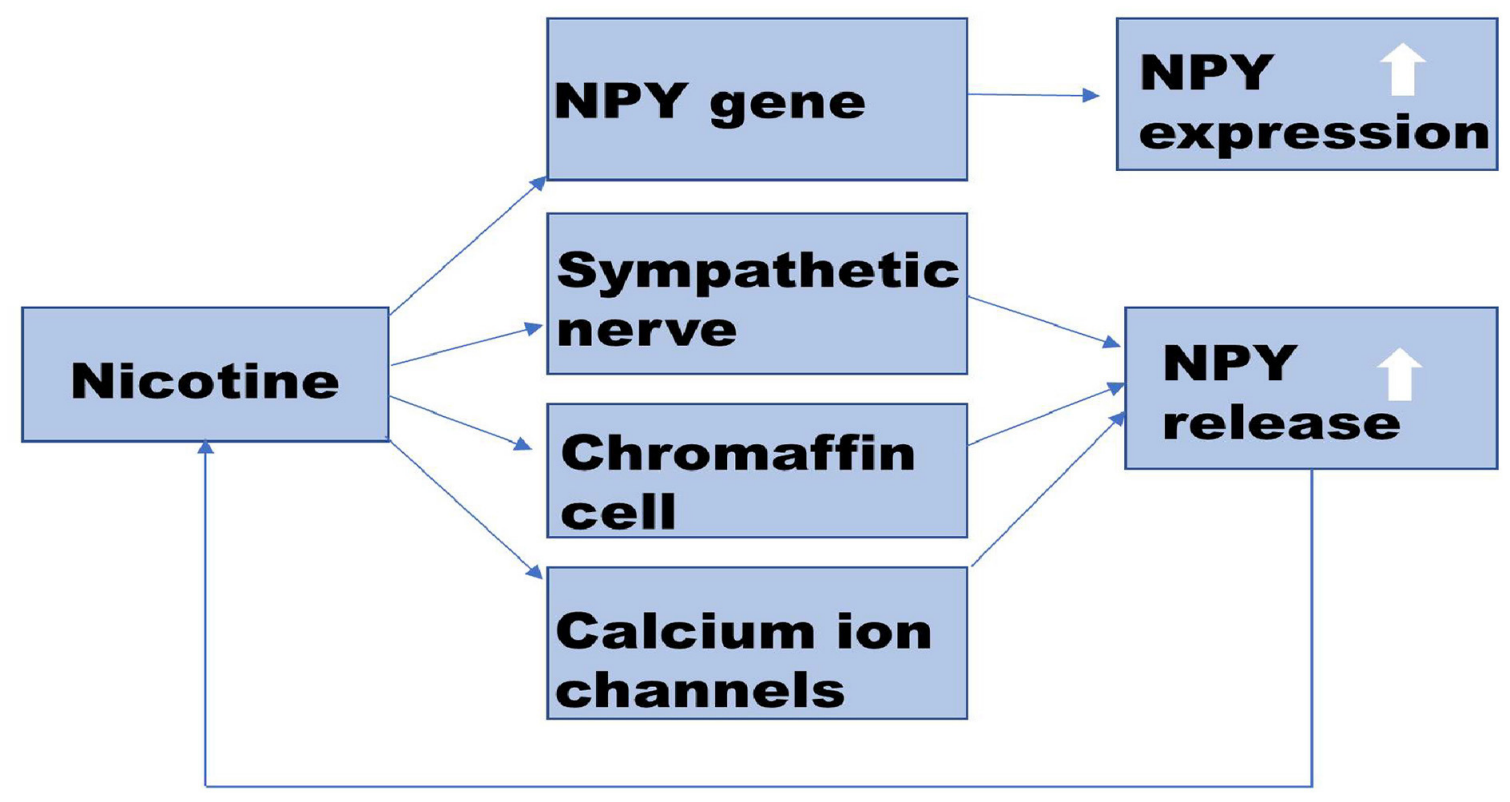
Potential mechanism of nicotine-promoting NPY expression. Nicotine promotes the synthesis and release of NPY. In the central nervous system, nicotine up-regulates the expression of the *NPY* gene. In the peripheral nervous system, nicotine promotes NPY release by stimulating nAChR on sympathetic nerve and chromaffin cells. Nicotine also can activate calcium channels on the cell membrane and promote the co-release of NPY with NA. Increased NPY can affect nicotine intake. NPY, Neuropeptide Y; nAChR, nicotinic acetylcholinergic receptor; NA, norepinephrine.

**Figure 3:**
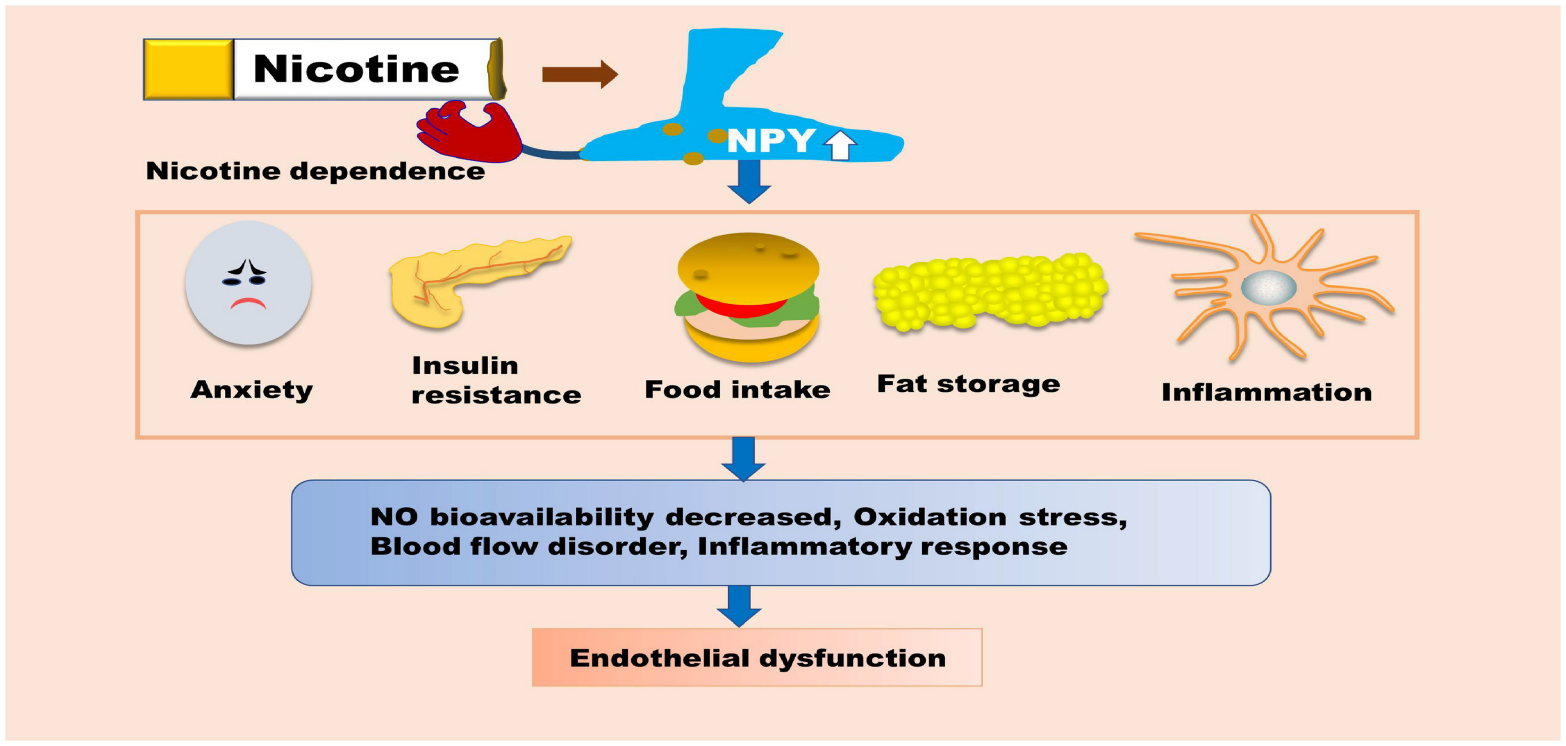
NPY is indirectly involved in nicotine-induced endothelial dysfunction. NPY can promote insulin resistance, food intake, and fat storage, as well as enhance macrophage-mediated inflammatory responses. NPY can also increase the body's dependence on nicotine, which indirectly aggravates endothelial dysfunction. These pathological processes can promote oxidative stress, inflammatory responses, blood flow disorders, and reduce the bioavailability of NO, ultimately inducing endothelial dysfunction. NPY, Neuropeptide Y; NO, nitric oxide.

Nicotine is widely believed to be involved in various pathophysiological processes that induce endothelial dysfunction in a dose and time-dependent manner, such as promoting vasoconstriction, inducing insulin resistance, stimulating oxidative stress, and disrupting anticoagulant and procoagulant systems. Administering nicotine during postnatal days 1–8 upregulates mRNA expression of NPY in the hypothalamus of neonatal rat pups (59). In the central, nicotine promotes the expression of NPY in the hypothalamus by up-regulating the *NPY* gene in the rat. In the peripheral, nicotine probably promotes the release of NPY from the rat heart and adrenal gland by regulating calcium channels. Besides, nicotine can promote the co-release of NPY and NA, by directly stimulating the sympathetic nerve, as is shown in Figure 2. Similar to its effects in animals, nicotine also increased the release of NPY in human adrenal chromaffin cells (60–62). Table 2 shows that nicotine promotes NPY expression, which in turn induces endothelial dysfunction. Herein, we critically reviewed the relationship between nicotine and the NPY system to provide a broad understanding of the pathophysiological mechanisms of nicotine-induced endothelial dysfunction, especially in AS (Figure 3).

**Table 2:** Relationship between NPY and nicotine.

**Nicotine administration**	**Location**	**Role**	**References**
4 mg/kg/days, 14 d	Hypothalamus (rat)	Enhances NPY expression and promotes food intake	(2)
6 mg/kg/days, 14 d	Hypothalamus (adult rat progeny)	Changes hypothalamic neuropeptides in the adult progeny	(3)
10 μmM/l, 10 min	Coronary venous (guinea pig)	Induces NA release and promotes NPY overflow	(4)
0.25, 1.5, and 3 mg/kg, twice daily, 8 days	Arcuate nucleus (neonatal rat pups)	Increases expression of NPY	(60)
100 μM, 10 min	Adrenal chromaffin cells (human)	Elicits a rapid increase in the release of NPY	(62)
5 mg/kg, 6 h	Adrenal (rat)	Upregulates neuropeptide synthesis	(63)

### NPY, Nicotine, and Vasoconstriction

It is generally accepted that nicotine enhances vasoconstriction by disrupting the balance between vasoconstrictors (such as NA) and vasodilators (such as NO), especially in coronary arteries with endothelial damage (63). As demonstrated, nicotine elevates the level of plasma NPY, both NPY and NA are powerful vasoconstrictors (64–67). There seems to be a consensus that NPY and NA are stored together in presynaptic vesicles and in large, dense-core vesicles. In addition, NPY and NA appear to be released together, although there is no conclusive evidence describing this phenomenon (68). Low concentrations of NPY directly affect vasoconstrictors in coronary arterial microvessels (69). Electrically evoking NPY release from the perivascular nerve terminals of breast vascular and radial artery biopsies showed that NPY performed synergistically with NA to modulate vascular tone and potentiated NA-mediated vasoconstriction (27, 39). This phenomenon may be precipitated by changes in NA levels mediated by NPY-induced sympathetic excitation (70, 71). In particular, NPY-induced TXA2 release may have a strong effect on NA-mediated vasoconstriction, further confirmed by studies inhibiting NPY spontaneous vasoconstriction with TXA2 synthase inhibitors (72). NPY evokes potent, long-lasting vasoconstriction by binding to Y1R on ECs and smooth muscle cells (40, 42) induced by sympathetic stimulation, but not NA (73). Long-term incubation with NPY decreases the expression of eNOS mRNA and eNOS protein levels in human umbilical vein endothelial cells (74). There is correlation between gender and NPY-induced vasoconstriction; the latter is reduced in the presence of female sex hormones (75). Therefore, we speculate that nicotine-induced elevated NPY disrupts the bioavailability of NO. Nicotine induces several cardiovascular effects, from increasing myocardial contractility and blood pressure to increasing cardiac load and blood flow resistance. The potential mechanism for this is nicotine stimulating the release of NPY and NA by activating nAChR localized on peripheral postganglionic sympathetic nerve endings and the adrenal medulla.

### NPY, Nicotine, and Angiogenesis

Angiogenesis is a vital pathophysiological process that includes the proliferation and migration of ECs and it is regulated by a series of stimuli (76–78). Indeed, abnormal angiogenesis can induce or augment pathological conditions. Nicotine plays a substantial role in the proliferation of vascular ECs and in pathological angiogenesis in ischemia (79, 80). Analogous to the effects of nicotine, NPY can promote EC proliferation and angiogenesis in atherosclerotic arteries (28, 81), which can increase the risk of AS. Platelet NPY stimulates EC mitosis through Y1R, and stimulates EC proliferation through Y2R and Y5R, thereby promoting plaque neovascularization (82). Besides, plaque neovascularization notably destabilizes plaque and increases risk of bleeding. Intimal thickening and plaque formation induced by nicotine leads to disturbances in blood flow patterns, with lowered net forward flow and shear stress. In contrast, NPY increases risk of re-infarction after angioplasty, and is an important contributor to ischemic tissue after angioplasty, as it promotes neointima, thrombosis, and vascular obstruction by activating Y1R and Y5R (29). Furthermore, Y1 and Y5 receptor inhibitors can reduce these pathological processes and suggest a potential target for the treatment and prevention of vascular plasticization-related complications (83). NPY increases angiogenesis and arteriogenesis, but does not increase blood flow to the ischemic myocardium (30). Therefore, we speculate that NPY directly correlates with nicotine-induced pathological EC proliferation and angiogenesis. However, the specific mechanism needs further study. Nicotine affects the secretion function of ECs, leading to platelet adhesion and aggregation, by increasing TF and TXA2 expression, reducing prostaglandin I-2 expression, and NPY combined with those to promote thrombosis. NPY not only directly stimulates vasoconstriction associated with platelet aggregation to promote thrombosis (84, 85), but also induces anxiety (31, 86), which contributes substantially to platelet activation (32) by activating Y2R in the hypothalamus and striatum. Therefore, nicotine and NPY play a combined role in promoting pathological angiogenesis and thrombosis.

### NPY, Nicotine, and Energy Metabolism

Smoking has been well-established as an independent risk factor for AS. Nicotine exposure can cause a variety of pathological effects on ECs, among which the disturbance of energy metabolisms of ECs is particularly destructive (87). Chronic nicotine consumption promotes the expression of NPY (88), which is involved in the regulation of energy metabolism (36, 89–91), and shows a gender-dependent difference in the hypothalamus (92). NPY is a powerful appetite peptide (93, 94). Elevated NPY levels in the hypothalamic arcuate nucleus lead to hyperphagia and significant body weight gain. It is also known that excess energy can result in hyperlipidemia and hyperglycemia, the latter of which can directly destroy protein structures, damaging blood vessels. Hyperlipidemia and hyperglycemia can both increase the production of inflammatory cytokines, which can prompt foam cell formation and induce endothelial dysfunction. However, nicotine can inhibit weight gain by increasing leptin expression in the hypothalamus of food-deprived rats (95). Evidence supports the notion that NPY plays an important role in inhibiting insulin secretion, causing hyperglycemia in mice (96). NPY binding to Y1R on islet cells induces insulin resistance and enhances beta-cell replication by regulating the extracellular signal activity (34). Moreover, hyperglycemia, caused by insulin resistance and abnormal insulin secretion, can lead to ROS accumulation and decreased NO bioavailability, thus promoting endothelial dysfunction (97–100).

### NPY, Nicotine, and Oxidative Stress

Nicotine is a powerful oxidant that increases ROS production in plasma and induces leukocyte adhesion. Both of these effects, if left uncontrolled, will lead to expanded intercellular space of ECs and endothelial dysfunction (101). Furthermore, nicotine not only destroys lipid homeostasis but also oxidizes blood lipids to lipoproteins of different densities via ROS, thus promoting leukocyte phagocytosis of lipoproteins and forming foam cells, which is the key pathogenesis of AS (102, 103). NPY promotes the storage of fats such as triglycerides and cholesterol and increases the source of lipoproteins, leading to oxidative stress and endothelial dysfunction (104). NPY and endothelial dysfunction can reinforce each other. Endothelial dysfunction can stimulate the secretion of NPY and promote leukocyte chemotaxis, thus expanding vascular inflammation (37, 105–107). NPY also directly regulates inflammation in human ECs (108, 109). In addition, inflammation stress plays a role in obesity-related cognitive impairment (110, 111). Lipid deposition induced by NPY and an increase in oxidative substances induced by nicotine can not only increase blood viscosity, but also promote the formation of lipid strips in AS.

### NPY and Nicotine Dependence

Nicotine addiction is a chronic disorder characterized by dysphoria upon nicotine withdrawal and relapse after periods of abstinence. Withdrawal and relapse increase levels of NPY and its receptor proteins in the central nervous system, especially the Y1 receptor associated with brain reward function (112). NPY and Y1R agonists improve pathological withdrawal signs and negative affective states (113). Conversely, increased Y2R expression in the hippocampal CA3 region might play an important role in nicotine withdrawal-induced social dysfunction behavior and is involved in the mediation of nicotine relapse (38). Importantly, manipulations of Y1R and Y2R signals can regulate nicotine usage and Y1R agonists and Y2R antagonists promote reduced nicotine intake in central system regions (114, 115). In addition, upon investigating 2517 Japanese elderly smokers, it was discovered that the prevalence of the *NPY2R* rs4425326 C allele and the rs4425326 homozygous T allelotype was obviously associated with nicotine dependence (116). Thus, NPY can affect nicotine consumption, and is a promising target for treating nicotine-induced endothelial dysfunction.

In conclusion, nicotine can regulate the expression of NPY, which can affect human nicotine intake. NPY may play a role as an enhancer in nicotine-induced endothelial dysfunction. Nicotine can cause changes in damage to the vascular wall, initiation of atherogenesis, hemorheological parameters, and coronary artery hemodynamics. NPY can increase blood flow resistance by promoting not only vasoconstriction but also platelet aggregation and vascular plaque formation. NPY can also promote lipid deposition, inflammatory reaction, and leukocyte adhesion, resulting in a hypercoagulable blood state. As such, NPY aggravates blood flow disorder induced by nicotine. The flow disordered can further induce endothelial dysfunction.

## Conclusions and Future Directions

The role of NPY polymorphism in the regulation of cardiovascular activity has been studied, but the effect on endothelial function has varied in different studies, possibly due to varied receptor effects. Few studies have explored the regulation of NPY in vascular endothelial dysfunction and AS. Lagraauw et al. observed that focalized NPY overexpression in the carotid artery significantly increased atherosclerotic plaque size and perivascular mast cell activity in apoE(–/–) mice. NPY may impact plaque progression in part via mast cell activation (28). In particular, the role of NPY in vascular endothelial dysfunction in smokers remains unclear. Polymorphisms of the *NPY* gene determine its functional complexity, reflected in its ability to induce angiogenesis and vascular remodeling. NPY improved functional blood flow in mice with hind limb ischemia (44), but has also contributed to the development of AS by promoting thrombosis and oxidative stress blood vessels (117). The discovery and complete utilization of NPY functions, including the promotion of EC proliferation and NO secretion, may direct future research and generate hope for the clinical treatment of arteriosclerotic cardiovascular disease. Evidence suggests that an excited sympathetic nervous system induced by an acute coronary heart attack, can promote NPY release, thereby causing coronary artery spasm and aggravating further myocardial ischemia. However, additional *in vitro* and *in vivo* experimental studies are urgently needed to further support these findings. NPY led to neointima formation, and triggered thrombosis and vessel occlusion. Therefore, NPY receptor antagonists may offer a new approach to treating restenosis. NPY demonstrated an important role in stem cell therapy for acute myocardial infarction, by regulating vascular access for progenitor cells (118), as well as defended the nerves of bone marrow (119, 120). NPY diversity determines the complexity of its functions. NPY can promote cell proliferation, increasing the risk of coronary heart disease reinfarction and rebleeding. Smoking can affect the expression of NPY, which can aggravate endothelial dysfunction and blood flow disorder induced by nicotine. In light of this, NPY receptor-targeted therapy may be useful in treating nicotine-related cardiovascular diseases. At present, research on NPY is mostly limited to animal experiments; therefore, more human experiments are needed to further confirm the function and mechanism of NPY. A thorough study on the relationship between NPY and coronary heart disease may open the door for new treatments for the latter. Moreover, the prevention and treatment of nicotine-related cardiovascular diseases present a major challenge for providing medical care.

## Author Contributions

Y-lZ helped to draft the manuscript and prepare tables and figures. W-dW and M-mL contributed to an extensive literature review. H-lL and SL provided the subject of the review, critically revised, and edited the manuscript. All authors have read and approved the final version of the manuscript, and agreed with the order in which the authors are presented.

## Conflict of Interest

The authors declare that the research was conducted in the absence of any commercial or financial relationships that could be construed as a potential conflict of interest.

## Abbreviations

NPY: Neuropeptide Y
AS: Atherosclerosis
ECs: Endothelial cells
EC: Endothelial cell
TXA2: Thromboxane A2
NO: Nitric oxide
NOS: Nitric oxide synthases
eNOS: Endothelial NOS
nNOS: Neuronal NOS
iNOS: Inducible NOS
TF: Tissue factor
VEGF: Vascular endothelial growth factor
nAChR: Nicotinic acetylcholinergic receptors
NA: Norepinephrine
ROS: Reactive oxygen species.
